# ﻿Establishing a *COI* DNA barcoding reference database for Taiwanese coastal fish species from egg and larvae specimens

**DOI:** 10.3897/zookeys.1247.151795

**Published:** 2025-07-30

**Authors:** Kwang-Tsao Shao, Hui-Ling Ko, Yung-Chieh Chiu, Hsin-Kai Huang, Ya-Fang Chen, Yen-Wei Chang

**Affiliations:** 1 Institute of Marine Biology, National Taiwan Ocean University, Keelung, Taiwan National Taiwan Ocean University Keelung Taiwan; 2 Marine Fisheries Division, Fisheries Research Institute, Ministry of Agriculture, Keelung, Taiwan Marine Fisheries Division, Fisheries Research Institute, Ministry of Agriculture Keelung Taiwan; 3 Seafood Technology Division, Fisheries Research Institute, Ministry of Agriculture, Keelung, Taiwan Fisheries Research Institute, Ministry of Agriculture Keelung Taiwan

**Keywords:** barcoding region, *COI*, DNA barcoding, DNA reference database, eDNA, fish eggs, fish larvae, species identification

## Abstract

Fish eggs and larval stages are essential components of marine ecosystems and play important roles in sustaining marine food webs. However, the egg and larval stages often lack distinct diagnostic characteristics, making it challenging to identify species solely based on their morphology. In this study, we applied a DNA barcoding approach targeting the cytochrome *c* oxidase subunit I gene to establish a comprehensive reference for fish eggs and larval material in coastal waters off Taiwan. A total of 7602 records of fish eggs and larvae were collected from marine coastal waters surrounding Taiwan between 2004 and 2023 and identified using a DNA barcoding approach. By comparing our newly generated DNA barcoding sequences with records from public reference databases, we identified 1112 different fish taxa encompassing 24 orders, 158 families, 500 genera, and 844 fish species. This DNA barcoding referencing effort will facilitate future studies on the early life stages of Taiwanese coastal marine fish communities. In addition, it will also provide an important baseline for the development of new methods, such as eDNA and DNA metabarcoding diet analysis. This database provides comprehensive spatial and temporal distribution data of fish eggs and larvae in the waters surrounding Taiwan, contributing to a better understanding of fish spawning seasons, spawning grounds, and resource fluctuations. This database enhances the accuracy of species identification and serves as a scientific foundation for biodiversity conservation and fishery resource management.

## ﻿Introduction

Understanding the early life history stages of fish, including eggs and larvae, is essential for advancing our knowledge of population dynamics (Chen et al. 2018; Jiang et al. 2024; Nahid et al. 2024), reproductive ecology (Hou et al. 2021a), and habitat utilization (de Lima et al. 2024). These stages provide critical information that cannot be obtained from adult samples, such as the spawning season, spawning ground location, and population replenishment. Fish eggs and larvae play vital roles in marine ecosystems and contribute to species diversity, fishery resource dynamics, and ecological conservation. However, due to the rapid morphological changes during development and the lack of distinct diagnostic characteristics at the species level, morphological convergence often occurs among taxa. Consequently, species identification based solely on morphology is highly challenging, with reported average accuracies of only 80.1% at the family level, 41.1% at the genus level, and 13.5% at the species level (Ko et al. 2013). This taxonomic uncertainty has limited our ability to connect the different life history stages of fish.

DNA barcoding was first proposed by Hebert et al. (2003) as a standardized method for species identification based on the sequencing of a short mitochondrial gene region, typically the cytochrome *c* oxidase subunit I (*COI*) gene (~650 bp). This technique has been widely applied in taxonomy, biodiversity conservation, environmental monitoring, and seafood traceability because of its high efficiency in distinguishing closely related species and detecting cryptic taxa (Ardura et al. 2016; Lim et al. 2016; Chang et al. 2019; Chu et al. 2019; Chiu et al. 2022). These applications provide valuable reference data for fishery resource management (Lin et al. 2016). Furthermore, the accuracy and comprehensiveness of adult fish barcode databases are essential for ensuring the reliable identification of early life stages, such as eggs and larvae (Hou et al. 2021b).

Since 2004, Taiwan has implemented the “Taiwan Barcode of Life” (TaiBOL) project, which aims to preserve frozen tissue samples and voucher specimens of native wildlife while establishing a digitized and publicly accessible database (http://cryobank.sinica.edu.tw). This initiative serves as a critical foundation for the conservation of Taiwan’s biodiversity resources and the assertion of national sovereignty over indigenous biological materials. Under the support of the TaiBOL project, Taiwan has developed a DNA barcode reference database for adult teleost fishes, covering over 40% of the native ray-finned fish species. This dataset, titled “Native Teleost Fishes in Taiwan,” has been uploaded to the Barcode of Life Data System (BOLD). It comprises 2993 individual records representing 29 orders, 184 families, 637 genera, and 1245 species of Actinopterygii, providing a robust and reliable foundation for species identification and comparative taxonomic research (Chang et al. 2017).

This study aimed to establish a DNA barcode reference database for fish eggs and larvae collected from the waters surrounding Taiwan, providing systematic genetic data to support species identification and ecological research. From 2004 to 2023, 7602 specimens were collected, forming the foundation for the publication of ‘Eggs and Larvae of 500 Taiwan Fishes’ (Shao et al. 2024), an atlas featuring DNA-verified images of 505 species across 29 orders and 121 families, including fish eggs and various developmental stages of larval fish. This book features photographs of early life history stages, serving as an essential morphological reference for future species identification, research, and educational purposes. The resulting database not only provides a foundation for investigating the early life history stages of fish in Taiwanese waters but also serves as a valuable resource for conservation and fisheries resource management. In addition, it complements environmental DNA (eDNA) research by validating analytical results, improving identification accuracy, and enhancing the interpretation of species spatiotemporal distribution during spawning seasons and larval occurrence. This integration strengthens the complementary applications of eDNA and traditional monitoring approaches.

## ﻿General description

**Figure 1.**
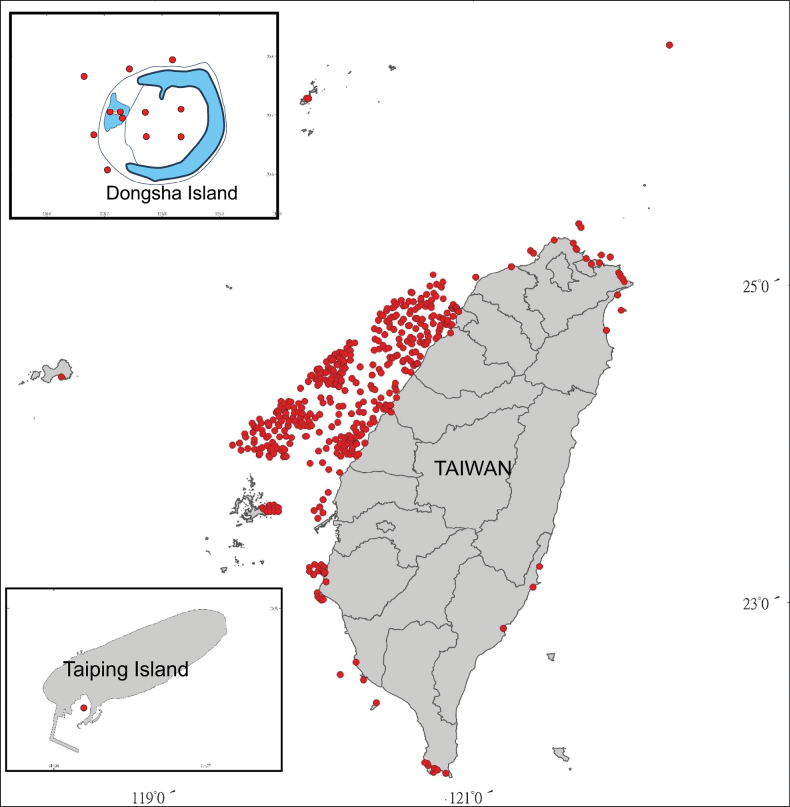
The distribution of fish eggs and larval fish sampling stations from the coastal waters off Taiwan.

This database of fish eggs and larvae, verified through DNA barcoding, was compiled by a research team led by Dr Kwang-Tsao Shao, who had retired from the Biodiversity Research Center at Academia Sinica. Specimens were collected from waters surrounding Taiwan between May 2004 and February 2023, from northern Taiwan to Taiping Island in the South China Sea. Most sampling stations were concentrated in the western waters of Taiwan (Fig. 1).

The ichthyoplankton samples were collected using plankton nets or modified light traps. The plankton nets had a mesh size of 330 µm, a mouth diameter of 90 cm, and a net length of 180 cm. Tows were conducted at the surface (~1 m depth) for 10 min per station, and a flowmeter (HydroBios) was mounted at the mouth of the net to quantify the volume of the filtered water. The light traps were modified based on the design proposed by Doherty (1987), with approximate dimensions of 30.7 × 30.7 × 61 cm, a weight of 8 kg. Each trap had multiple elongated entrance openings on its sides to facilitate the entry of fish larvae, as described by Chan et al. (2016). Light traps were deployed at night in coastal or harbor waters for approximately 4–5 h. The number of traps deployed at each sampling station ranged from one to two, depending on the sampling session and environmental conditions.

Subsequently, the fish eggs and larvae were selected and preserved in 95% ethanol. Under a dissecting microscope, preliminary morphological identification of fish eggs and larvae was conducted following identification guidelines (Okiyama 1988; Neira et al. 1998). The specimens were categorized into different types (e.g., Type 1 and Type 2) based on their distinguishing features. The egg diameter and standard length were measured, and photographs were taken.

DNA was extracted from the eggs and larvae of individual fish. The *COI* barcoding procedure followed protocols described by Ko et al. (2013) and Chang et al. (2016). Genomic DNA was extracted using either the Genomic DNA Mini Kit (Geneaid, Taiwan) or the QuickGene DNA Tissue Kit S (KURABO, Japan). A ~650 bp fragment of the mitochondrial *COI* gene was amplified via polymerase chain reaction (PCR) using the primer pair FishF1 and FishR1, modified from Ward et al. (2005). Each 25 μL PCR reaction contained 17.9 μL ultrapure water, 2.5 μL 10× PCR buffer, 0.3 μL dNTPs (40 mM), 1 μL of each primer (1 μM), 0.3 μL Taq DNA polymerase, and 2 μL DNA template. PCR thermal cycling conditions were as follows: initial denaturation at 94 °C for 4 min, followed by 35 cycles of denaturation at 94 °C for 30 s, annealing at 47 °C for 30 s, and extension at 72 °C for 30 s, with a final extension at 72 °C for 7 min. The PCR products were examined using 1% agarose gel electrophoresis, and samples with clear bands were selected for purification and sequencing using MISSION BIOTECH (Taiwan). The resulting sequences were aligned using Clustal W and subsequently trimmed, assembled, and saved in FASTA format using BioEdit version 7.2.5 (Hall 1999).

All DNA sequences were analyzed in 2024 using the National Center for Biotechnology Information (NCBI) and BOLD databases for species identification. The taxonomic names and hierarchical classification used in this study followed the system adopted by FishBase (Froese and Pauly 2024). They were cross-referenced with the Catalog of Fishes maintained by the California Academy of Sciences to ensure consistency with internationally recognized fish classification standards. Specimens exhibiting a similarity of over 99% were identified at the species level, whereas those with a similarity between 97% and 98.99% were identified at the genus level (Ratnasingham and Hebert 2007). Molecular identification techniques were applied to classify fish eggs and larvae to the species or genus level whenever feasible. When multiple candidate species names were returned from the sequence comparison, we screened the reference *COI* sequences of adult fish within the same family that are known to occur in Taiwan. Unidentified egg or larval fish sequences were included alongside these references to construct a phylogenetic tree using the neighbor-joining method implemented in MEGA 11 software (Tamura et al. 2021). Kimura 2-parameter genetic distances were calculated to determine the most likely adult species.

**Figure 2.**
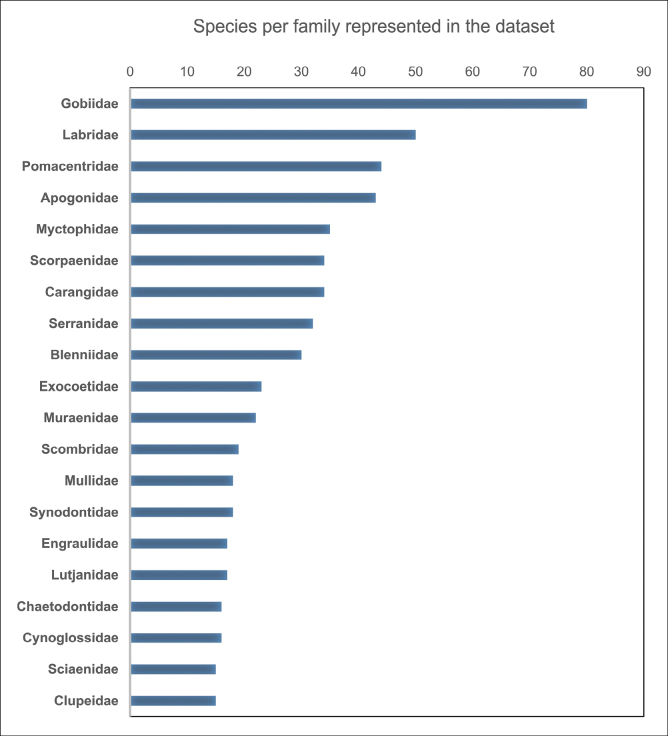
The top 20 families with the highest taxa in the fish egg and larval fish database from the coastal waters off Taiwan.

This database includes information on fish eggs and larvae, collection date, geographic location, coordinates, collection methods, and taxonomic classification obtained through comparison with the NCBI (Camacho et al. 2023) and BOLD (Ratnasingham and Hebert 2007; Ratnasingham et al. 2024) databases, including order, family, and species names. A total of 7602 sequences were obtained, including 3166 fish egg sequences and 4436 fish larval sequences, with a total of 1112 taxa encompassing 24 orders, 158 families, 500 genera, and 844 species. Among the sequence data, 267 taxa could not be identified at the species level, while 57 were identified at the family level and 210 were identified at the genus level. Among these 158 families, the top 20 families with the highest species diversity comprised 578 taxa, accounting for 51.98% of the entire database. According to the Fish Database of Taiwan (https://fishdb.sinica.edu.tw/), 3535 fish species have been recorded in Taiwanese waters (Shao 2025). In the present study, 844 fish eggs and larvae were identified to the species level using DNA barcoding, representing approximately 23.9% of the known fish fauna in Taiwan. The Gobiidae family was the most species-rich in the database, comprising 80 taxa; the Labridae followed it with 50 taxa, Pomacentridae with 44 taxa, and Apogonidae with 43 taxa (Fig. 2).

## ﻿Data published through

Taiwan Biodiversity Information Facility (TaiBIF): https://portal.taibif.tw/

### ﻿Taxonomic ranks

**Kingdom**: Animalia.

**Phylum**: Chordata.

**Class**: Actinopterygii.

**Order**: Anguilliformes, Atheriniformes, Aulopiformes, Beloniformes, Beryciformes, Clupeiformes, Cyprinodontiformes, Elopiformes, Gadiformes, Gasterosteiformes, Gonorynchiformes, Lampridiformes, Lophiiformes, Mugiliformes, Myctophiformes, Ophidiiformes, Osmeriformes, Perciformes, Pleuronectiformes, Scorpaeniformes, Siluriformes, Stomiiformes, Tetraodontiformes, Zeiformes

**Family**: Acanthuridae, Acropomatidae, Ambassidae, Ammodytidae, Antennariidae, Aploactinidae, Apogonidae, Ariidae, Atherinidae, Aulopidae, Aulostomidae, Bagridae, Balistidae, Banjosidae, Belonidae, Blenniidae, Bothidae, Bramidae, Bregmacerotidae, Bythitidae, Caesionidae, Callanthiidae, Callionymidae, Caproidae, Caracanthidae, Carangidae, Centriscidae, Centrolophidae, Cepolidae, Chaenopsidae, Chaetodontidae, Champsodontidae, Chanidae, Cheilodactylidae, Chirocentridae, Chlorophthalmidae, Cirrhitidae, Clupeidae, Congridae, Coryphaenidae, Creediidae, Cynoglossidae, Dactylopteridae, Diodontidae, Drepaneidae, Echeneidae, Eleotridae, Elopidae, Emmelichthyidae, Engraulidae, Ephippidae, Epigonidae, Exocoetidae, Fistulariidae, Gempylidae, Gerreidae, Gigantactinidae, Gobiesocidae, Gobiidae, Gonorynchidae, Gonostomatidae, Haemulidae, Hemiramphidae, Hexagrammidae, Holocentridae, Isonidae, Istiophoridae, Kraemeriidae, Kuhliidae, Kyphosidae, Labridae, Lampridae, Latidae, Leiognathidae, Lethrinidae, Lobotidae, Lutjanidae, Macrouridae, Malacanthidae, Megalopidae, Melanocetidae, Menidae, Monacanthidae, Monocentridae, Monodactylidae, Moronidae, Mugilidae, Mullidae, Muraenesocidae, Muraenidae, Myctophidae, Nemipteridae, Nettastomatidae, Nomeidae, Oneirodidae, Ophichthidae, Ophidiidae, Opistognathidae, Oplegnathidae, Ostraciidae, Paralepididae, Paralichthyidae, Parazenidae, Pempheridae, Percichthyidae, Percophidae, Peristediidae, Phosichthyidae, Pinguipedidae, Platycephalidae, Plecoglossidae, Pleuronectidae, Plotosidae, Poeciliidae, Polynemidae, Pomacanthidae, Pomacentridae, Priacanthidae, Pristigasteridae, Psettodidae, Pseudochromidae, Ptereleotridae, Rachycentridae, Regalecidae, Samaridae, Scaridae, Scatophagidae, Sciaenidae, Scombridae, Scombrolabracidae, Scombropidae, Scorpaenidae, Serranidae, Siganidae, Sillaginidae, Soleidae, Sparidae, Sphyraenidae, Sternoptychidae, Stomiidae, Stromateidae, Synaphobranchidae, Syngnathidae, Synodontidae, Terapontidae, Tetragonuridae, Tetraodontidae, Trachichthyidae, Trachipteridae, Triacanthidae, Triacanthodidae, Trichiuridae, Trichonotidae, Triglidae, Tripterygiidae, Uranoscopidae, Zanclidae, Zeidae.

### ﻿Datasets

#### ﻿Dataset description

This research project was led by Research Fellow Kwang-Tsao Shao (retired) from the Biodiversity Research Center, Academia Sinica. The research team collected and compiled a database of fish eggs and larvae, which were identified using DNA barcoding. The dataset includes the following fields: eventDate, individualsCount, longitude, latitude, recordedBy, identifiedBy, lifeStage, scientificName, kingdom, phylum, class, order, and family.

The specimen collection period spanned May 2004 to February 2023. It covered the surrounding waters of Taiwan, ranging from the northern waters of Taiwan to the Taiping Island waters in the South China Sea. Most sampling stations were concentrated in the western waters of Taiwan.

- **Object name**: Taiwan Fish Eggs and Larvae DNA Barcode Database
- **Character encoding**: UTF-8
- **Format name**: Darwin Core Archive format
- **Format version**: 1.0
- **Distribution**: https://ipt.taibif.tw/resource?r=taiwanfishlarvaedb
- **Publication date of data**: 2025-07-11
- **Language**: English
- **Metadata language**: English
- **Date of metadata creation**: 2025-07-11
- **Hierarchy level**: Dataset

### ﻿Copyright and usage guidelines

We kindly request that you adhere to the data usage guidelines. When using the data for research purposes, please notify the database author, Research Fellow Kwang-Tsao Shao.

## ﻿Additional information

### Use of AI

No use of AI was reported.

### Author ORCIDs

Kwang-Tsao Shao https://orcid.org/0000-0002-4807-7539

Yung-Chieh Chiu https://orcid.org/0000-0002-6565-596X

Yen-Wei Chang https://orcid.org/0000-0003-2164-4924

### Data availability

All of the data that support the findings of this study are available in the main text.
